# Diffusion weighted imaging abnormalities and cerebral ischemia in a cohort of patients on lecanemab

**DOI:** 10.1002/alz.71392

**Published:** 2026-04-21

**Authors:** Dylan Ryan, Michael W. Lutz, Tori Sides, Richard O'Brien, Kim Johnson

**Affiliations:** ^1^ Department of Neurology Duke University School of Medicine Durham North Carolina USA

**Keywords:** amyloid‐related imaging abnormalities, cerebral amyloid angiopathy, diffusion weighted imaging, lecanemab, stroke

## Abstract

**INTRODUCTION:**

We evaluate the frequency of diffusion weighted imaging abnormalities consistent with stroke in patients on lecanemab, focusing on the association between amyloid‐related imaging abnormalities (ARIAs) and stroke incidence.

**METHODS:**

For this retrospective cohort study, data were collected from time of lecanemab initiation to last follow‐up. Descriptive statistics characterized demographic and clinical factors for patients who experienced interval stroke in contrast to individuals who did not experience stroke. Propensity score matching was performed to evaluate the association between ARIAs and stroke accounting for confounding factors.

**RESULTS:**

Of 261 patients, 11 (4.2%) were diagnosed with interval stroke. Stroke patients were older (80.1 vs. 74.3 years had lower median Montreal Cognitive Assessment cognitive scores (19 vs. 22) and higher prevalence of ARIAs (54.5% vs. 20.8%). A significant association (*P* = 0.011) was observed between ARIAs and stroke incidence after propensity score matching. Strokes were small, asymptomatic, and mostly cerebellar (36.4%) or cortical (36.4%).

**DISCUSSION:**

Diagnosis of an ARIA was significantly associated with increased stroke. Higher than expected rates of cerebellar and cortical stroke and relationship to ARIAs suggests a possible common, immune‐mediated mechanism between these regions of restricted diffusion and ARIAs. Further study is warranted.

## Highlights

- Interval ischemic lesions occurred in 4.2% of patients on lecanemab in a single‐center study.
- There was a significant association with a history of amyloid‐related imaging abnormalities (ARIAs) and development of interval ischemic lesions in patients on lecanemab that remained after propensity score matching.
- Temporal association, the rate of cortical and cerebellar regions, and the significant association with ARIAs may suggest an immune‐mediated process in regions with high amyloid burden causing local ischemia.

## 1 INTRODUCTION

As the population ages in the United States, studies project the estimated cases of newly diagnosed dementia will rise to 1 million people per year by 2060.^1^ The most common cause of dementia worldwide and in the United States is Alzheimer's disease (AD), which accounts for ≈ 60% to 70% of cases with an incidence that increases after age 65.^2^ Given that AD is a progressive disease and a leading cause of domestic disability, there has been continued need for disease‐modifying therapies.

The development of anti‐amyloid immunotherapies (AATs), with lecanemab and donanemab receiving US Food and Drug Administration (FDA) approval, has led to a paradigm shift in the management of patients with mild cognitive impairment (MCI) and mild dementia in patients with positive AD biomarkers. These therapies are becoming more common but do come with management challenges. Patients with AD treated with AATs are monitored with magnetic resonance imaging (MRI) for changes due to treatment including brain edema or bleeding events termed amyloid‐related imaging abnormalities (ARIAs). ARIA‐E (edema) refers to vasogenic edema secondary to blood–brain barrier disruption while ARIA‐H (hemorrhage) represents microhemorrhages, macrohemorrhages, and superficial siderosis that can lead to symptomatic intracranial hemorrhage (sICH).^3^ These changes are hypothesized to be secondary to antibody binding to amyloid within the vasculature, leading to changes in vessel integrity causing vasogenic edema and hemorrhage.^4^ Approximately 80% of cases of ARIA are asymptomatic,^5^ and incidence of severe ARIAs is ≈ 3.5%,^6^ although rates do increase with the presence of one or two copies of the apolipoprotein E (*APOE*) ε4 allele.^7^, ^8^, ^9^

In addition to ARIAs, patients with AD are also at an increased risk of cerebrovascular disease. Patients with AD often possess vascular risk factors, such as hypertension and hyperlipidemia, which can further contribute to cognitive decline. Approximately one third of patients presenting with new ischemic stroke are over age 80,^10^ and many of these patients have comorbid AD pathology.

Less is known about the frequency, pattern, risk factors, and management of patients on AATs with diffusion abnormalities on MRI that are consistent with stroke. In this single‐center, observational cohort study we describe the frequency, characteristics, associated risk factors, and management of patients with MRI diffusion abnormalities consistent with stroke in the setting of lecanemab use.

## 2 METHODS

### 2.1 Standard protocol approvals, registrations, and patient consents

This study is a retrospective analysis of patients with AD‐related MCI and mild dementia managed on lecanemab at the Duke University Memory Disorders Clinic. The study was approved by the Duke Institutional Review Board. Patients had positive cerebrospinal fluid (CSF) AD biomarkers using amyloid beta (Aβ) 42/40 ratio and/or phosphorylated tau to Aβ42 ratio, and/or positive amyloid positron emission tomography (PET) prior to use of lecanemab. Informed consent for treatment with lecanemab was previously obtained from the participants. Lecanemab was given according to appropriate use recommendations^11^ with biweekly infusions with planned MRI monitoring performed prior to the 5th, 7th, and 14th infusions or if patients had symptoms suggestive of potential ARIA. There was a uniform dosing protocol within the center for patients receiving lecanemab.

### 2.2 Study population

We included all patients at the Duke University Memory Disorders Clinic receiving lecanemab. No patients were excluded based on missing clinical data and analyses were performed based on available data within the electronic medical record and our patient registry. Patient charts were reviewed using the electronic medical record. Demographic information including age at time of initiation of lecanemab, sex, race (White, Black, Hispanic, Asian, unknown/unspecified), diagnosis (MCI, AD), and presence of *APOE* ε4 alleles were collected from review. Variables related to diagnosis and lecanemab use included baseline cognitive scores, CSF and amyloid PET results, lecanemab initiation date, and interval follow‐up imaging dates. Diagnosis of ARIA was determined based on review of imaging by neuroradiologists at the academic medical center and review of imaging based on their reports. Diagnosis of ARIA‐E and ARIA‐H and radiographic severity corresponded to descriptions within the appropriate use recommendations of lecanemab.^11^ ARIA‐E appears on fluid‐attenuated inversion recovery (FLAIR) imaging on MRI as parenchymal white matter and sulcal hyperintensities with associated vasogenic edema,^12^ which is typically diffusion weighted imaging (DWI) negative.^13^ ARIA‐H included cerebral microbleeds, convexity subarachnoid hemorrhage, and cortical superficial siderosis on susceptibility‐weighted imaging (SWI) and/or T2* gradient recalled echo sequences.^12^, ^13^

**RESEARCH IN CONTEXT**

1. **Systematic review**: Previous randomized trials assessing use of anti‐amyloid immunotherapies (AATs) have primarily focused on amyloid‐related imaging abnormalities (ARIAs) as a primary safety signal. A review of case reports has revealed isolated instances of small, acute infarcts on diffusion weighted imaging within this population. This study used a retrospective cohort of 261 patients on lecanemab to assess these diffusion abnormalities to assess associated predictors and management of these patients.
2. **Interpretation**: Our findings showed 4.2% of patients were found to have developed interval ischemic lesions, all clinically silent. There was a significant association between ARIAs and these interval ischemic lesions that remained similar with propensity matching. These lesions were most commonly cortical and cerebellar, suggesting a possible link between vascular amyloid deposition, ARIAs, and focal ischemia in patients managed with AATs.
3. **Future directions**: Prospective registry studies could aid in determining the incidence, temporal dynamics, and clinical consequences of these lesions given the association between interval ischemia and ARIAs found within this study. This could aid in clarifying whether these lesions represent a distinct mechanism of vascular injury related to AAT use.

In this study, neuroradiology reports from patients treated with lecanemab were systematically reviewed. Cases in which the neuroradiologist interpreted imaging findings as consistent with stroke were subsequently independently reviewed by a board‐certified vascular neurologist. Patients were classified as having stroke only when this adjudication concurred with the neuroradiology interpretation. Inter‐rater agreement was 91.7%. Date of stroke and location of stroke were gathered through review of individual imaging and imaging documentation. Associated vascular risk factors were collected through review of the patient's electronic medical record with listed medical history at the time of the encounter. History of stroke and transient ischemic attack (TIA) was based on clinical record review and silent infarcts noted on imaging reports prior to initiation of lecanemab. Follow‐up medication information, vascular imaging, echocardiography, and cardiac monitoring was individually reviewed through the electronic medical record. Time between initiation of lecanemab and diagnosis of stroke was collected.

### 2.3 Statistical analysis

Descriptive statistics were calculated for patients’ characteristics, including clinical and comorbidity data for the overall sample, groups defined by the presence and absence of stroke. The demographic and clinical variables were analyzed to identify factors to include in the propensity score calculation for testing the association between ARIA and stroke and to characterize differences between the patients who experienced stroke and those that did not experience stroke. For age, a Wilk–Shapiro test for normality was performed followed by a *t* test with a Welch correction. For ordinal variables (Montreal Cognitive Assessment [MoCA], Mini‐Mental State Examination, Fazekas score), we conducted ordinal logistic regression. Categorical variables with two categories were tested for group differences for the presence/absence of stroke using a two‐tailed Fisher exact test. For *APOE* genotype, number of *APOE* ε4 alleles was used for statistical analysis. For *APOE* genotype and diagnosis (MCI, AD) a likelihood ratio chi‐squared test is used to compare frequencies between the stroke/no stroke groups. False discovery rate (FDR) *P* values were calculated to account for the multiple testing of the 17 variables compared between the groups. FDR‐adjusted *P* < 0.05 was considered statistically significant.

To test the association between ARIAs and stroke in patients receiving lecanemab, logistic regression after propensity score matching was performed to mitigate the difference between the number of individuals experiencing stroke versus no stroke and adjust for potential confounders including age, sex, *APOE* genotype, baseline MoCA, and diagnosis (MCI vs. AD). Matching ratio was 1:1 using the greedy nearest neighbor algorithm without replacement and a caliper setting of 0.25. A standardized mean difference < 0.1 was considered acceptable covariate balance for the propensity score matching. The balance of covariates post‐matching was examined.

## 3 RESULTS

A total of 261 patients receiving lecanemab were included within the study population. Baseline characteristics can be found in Table 1. A total of 11 patients (4.2%) were diagnosed with interval stroke after starting lecanemab with 1 patient (9.1%) later being diagnosed with a recurrent stroke after cessation of lecanemab. Patients with stroke had higher mean age compared to patients with no stroke as well as lower median baseline MoCA scores. There was a lower percentage of female patients in the stroke group compared to the no stroke group. There were similar rates of diagnosis of MCI, AD, and vascular risk factors between the two groups. There was a higher frequency of diagnosis of ARIAs in the stroke group (54.5%) compared to the no stroke group (20.8%). Statistical comparisons of the demographic and clinical variables used to identify factors to include for propensity score matching are provided in Table S1 in supporting information.

**TABLE 1:** Baseline characteristics of patients with stroke on lecanemab.

	No stroke (*N* = 250)	Stroke (*N* = 11)	Entire cohort (*N* = 261)
**Age**, mean (SD)	74.3 (6.3)	80.1 (3.6)	74.5 (6.4)
**Sex**, *n* (%)			
Female	133 (53.2%)	3 (27.3%)	136 (52.1%)
**Race**, *n* (%)			
White	230 (92.0%)	9 (81.8%)	239 (91.6%)
Black	9 (3.6%)	2 (18.1%)	11 (4.2%)
Other	11 (4.4%)	0 (0.0%)	11 (4.2%)
ε4 **alleles**, *n* (%)			
0	80 (32.0%)	5 (45.4%)	85 (32.6%)
1	140 (56.0%)	6 (54.5%)	146 (55.9%)
2	30 (12.0%)	0 (0.0%)	30 (11.5%)
**Diagnosis**, *n* (%)			
MCI	166 (66.4%)	8 (72.7%)	174 (66.7%)
AD	78 (31.2%)	3 (27.3%)	81 (31.0%)
**Baseline MoCA**, median (MAD)	22 (2)	19 (2)	22 (3)
**Baseline MMSE**, median (MAD)	26 (2)	26 (2)	26 (2)
**Fazekas score**, median (MAD)	1 (0)	1 (1)	1 (0)
**Any ARIA**, *n* (%)	52 (20.8%)	6 (54.5%)	58 (22.2%)
**Hypertension**, *n* (%)	136 (54.4%)	6 (54.5%)	142 (54.4%)
**Hyperlipidemia**, *n* (%)	178 (71.2%)	10 (90.9%)	188 (72.3%)
**Diabetes**, *n* (%)	40 (16.0%)	0 (0.0%)	40 (15.3%)
**Atrial fibrillation**, *n* (%)	15 (6.0%)	2 (18.1%)	17 (6.5%)
**BMI ≥ 30**, *n* (%)	56 (22.4%)	2 (18.1%)	58 (22.2%)
**Obstructive sleep apnea**, *n* (%)	75 (30.0%)	2 (18.1%)	77 (29.5%)
**Tobacco use**, *n* (%)	80 (32.0%)	4 (36.4%)	84 (32.2%)
**History of CVA/TIA**, *n* (%)	27 (10.8%)	1 (9.1%)	28 (10.7%)

Abbreviations: AD, Alzheimer's disease; ARIA, amyloid‐related imaging abnormality; BMI, body mass index; CVA, cerebrovascular accident; DWI, diffusion weighted imaging; MAD, mean absolute deviation; MCI, mild cognitive impairment; MMSE, Mini‐Mental State Examination; MoCA, Montreal Cognitive Assessment; SD, standard deviation; TIA, transient ischemic attack.

To account for possible confounders and differences in group sizes, propensity score matching was performed using the variables age, sex, *APOE* genotype (number of ε4 alleles), MoCA at baseline, and diagnosis. The propensity score matching provided appropriate balance between the stroke and non‐stroke groups, with a 91.8% reduction in standardized mean differences between the groups to a standardized mean difference of 0.09. Examination of the covariates post‐matching confirmed strong balance: age for the stroke group, mean age = 80.1 years (standard deviation [SD] = 3.6), for non‐stroke mean age = 80.3 years (SD = 7.5); sex for the stroke group 27% female, for non‐stroke 10% female; diagnosis for stroke group 27% AD, 73% MCI, for the non‐stroke group 36% AD, 64% MCI; baseline MoCA for stroke group median = 19, mean absolute deviation (MAD) = 2, for non‐stroke group median = 19, MAD = 1; *APOE* for both groups, 45% were *APOE* ε4 non‐carriers and 55% were ε4 carriers. For the propensity score matched analysis, the association between ARIA and stroke was significant (*p* = 0.011, Fisher's Exact Test, two‐tailed).

**FIGURE 1:**
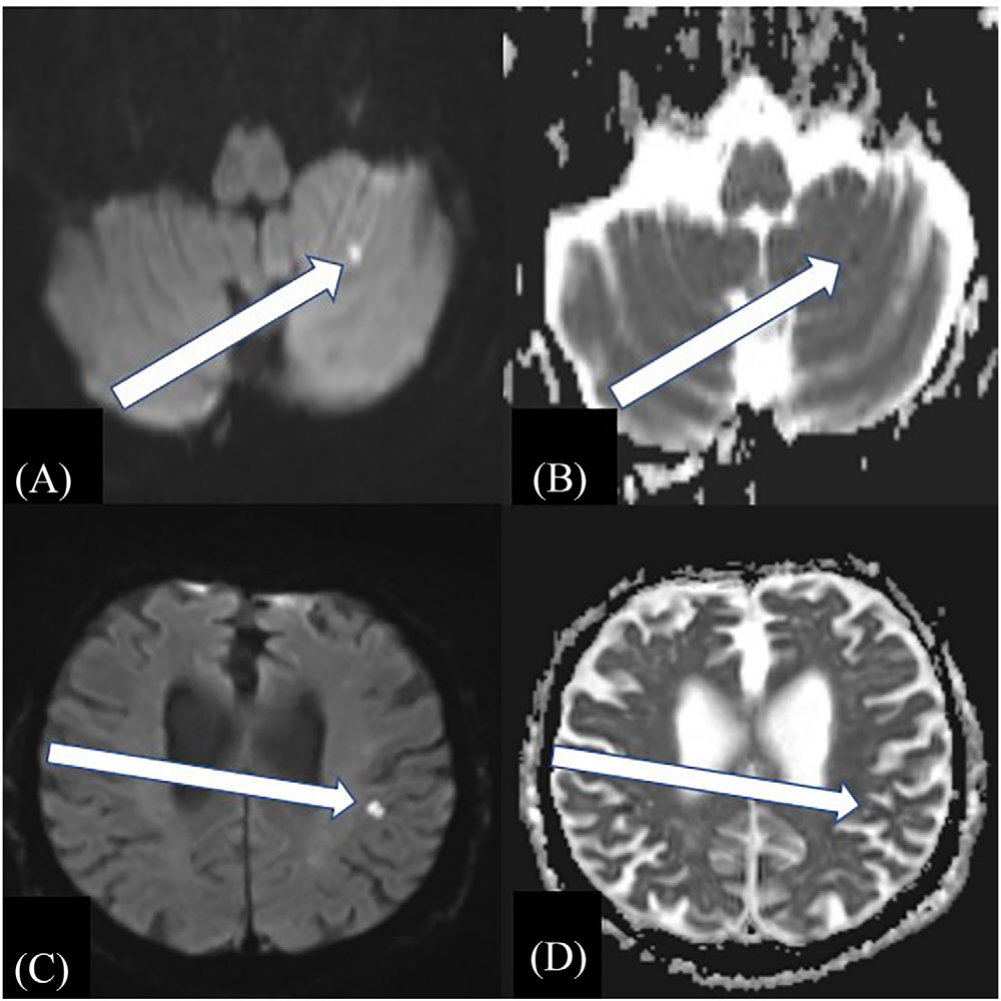
MRI imaging showing an area of hyperintensity on DWI imaging (A) with correlate on ADC imaging (B) consistent with an area of acute ischemic in the left cerebellum. MRI imaging showing an area of hyperintensity on DWI imaging (C) with correlate on ADC imaging (D) consistent with a cortical region of infarct in the left parietal lobe. ADC, apparent diffusion coefficient; DWI, diffusion weighted imaging; MRI, magnetic resonance imaging.

Characteristics of patients with stroke can be found in Table 2. Median time to stroke was 78 days from initiation of lecanemab. Regarding stroke location and size, four patients (36.4%) were diagnosed with cerebellar stroke and four patients (36.4%) were diagnosed with cortical infarcts (Figure 1). None of the patients experienced clinically localizable symptoms related to the imaging‐diagnosed strokes. Eight patients (72.7%) had vascular imaging performed with one patient having occlusive disease, though this was not in the territory of the infarct. Echocardiography was performed in seven patients (63.6%), with two patients (28.6%) having the finding of a patent foramen ovale. Extended cardiac monitoring was performed in eight patients (72.7%) with two patients (25.0%) having atrial fibrillation post stroke. Ten patients (90.9%) were managed with antiplatelet medications, one patient (9.1%) was started on anticoagulation, and one patient (9.1%) had a left atrial appendage closure. In all patients experiencing stroke, lecanemab infusions were stopped after diagnosis of stroke.

**TABLE 2:** Stroke imaging characteristics, workup, and management.

	Stroke (*N* = 11)
**Age at diagnosis**, median (IQR)	78 (42–86)
**Number of infusions**, median (IQR)	6 (4–15)
**Side of stroke**, *n* (%)	
Left	7 (63.6%)
Right	3 (27.3%)
Bilateral	1 (9.1%)
**Location of stroke**, n (%)	
Frontal lobe	3 (27.3%)
Parietal lobe	1 (9.1%)
Temporal lobe	2 (18.2%)
Occipital lobe	0 (0.0%)
Cerebellum	4 (36.4%)
Thalamus	1 (9.1%)
Basal ganglia	1 (9.1%)
Cortical	4 (36.4%)
**Vessel imaging completed**, *n* (%)	8 (72.7%)
**Echocardiogram performed**, *n* (%)	7 (63.6%)
Patent foramen ovale, *n* (%)	2 (28.6%)
**Extended cardiac monitoring performed**, *n* (%)	8 (72.7%)
Atrial fibrillation detected, *n* (%)	2 (25.0%)
**Secondary prevention**, *n* (%)	
Antiplatelet	10 (90.9%)
Anticoagulant	1 (9.1%)
**Left atrial appendage occlusion**, *n* (%)	1 (9.1%)

Abbreviation: IQR, interquartile range.

## 4 DISCUSSION

The use of AATs in patients with positive AD biomarkers is increasing, and clinical trials continue for expanding the use of this medication class. A known complication of these therapies is ARIAs, which can contribute to acute neurologic complications, but restricted diffusion on DWI was not a reported imaging abnormality with AATs in the clinical trials.^7^, ^14^, ^15^, ^16^ Within our cohort, 4.2% of patients developed an interval region of ischemia with restricted diffusion on DWI imaging on MRI and a history of ARIAs was significantly associated with increased risk of ischemia.

Inclusion of DWI imaging is recommended for drug monitoring in patients receiving AATs,^17^ as this sequence can aid clinicians in differentiating between ARIAs and acute ischemia. This distinction becomes important given treatment differences, including initiation of antithrombotics in patients with stroke and obtaining further vascular and cardiac imaging, whereas management of ARIA involves serial monitoring with consideration of corticosteroid use in severe cases. In our case, none of these interval ischemic events led to clinical symptoms but did lead to further vascular workup with initiation of antithrombotic therapy, and cessation of use of lecanemab.

The prevalence of silent brain infarction in patients increases over the age of 70^18^ and is more commonly seen in patients who undergo routine cerebral imaging. In addition, cerebral amyloid angiopathy (CAA) is associated with silent brain infarction in the geriatric population.^19^ Silent brain infarctions, though not presenting with clinical recognizable stroke syndromes, are associated with future risk of stroke and cognitive decline.^20^ Silent brain infarction rarely demonstrates restricted diffusion;^21^, ^22^, ^23^ thus, most appear chronic and are most commonly seen in the basal ganglia and subcortical white matter, and less often in the cortex and cerebellum.^18^ Within our cohort, among those with restricted diffusion on DWI consistent with ischemia, 36.4% of patients had a region of restricted diffusion in the cerebellum and 36.4% of patients had cortical involvement, which is atypical of common patterns of silent brain infarction.

The stroke pathology of our cohort, with cerebellar and cortical predominance, is most consistent with involvement of the superficial leptomeningeal cerebral and cerebellar arteries. There have been case reports of similar patterns of restricted diffusion in patients with ARIAs^24^, ^25^, ^26^ who had punctate areas of restricted diffusion on DWI, including in the cerebellum.^25^ Neuropathological examination shows that CAA is a vascular condition characterized by Aβ deposition in the vascular walls of cerebellar meningeal and cerebral cortical vessels.^27^ While the cerebellum is often used as a reference region on amyloid PET owing to its presumed relative sparing,^28^ superficial cerebellar microbleeds have been shown to correlate closely with CAA,^29^ suggesting that cerebellar vessels are not immune to amyloid‐related pathology. Lecanemab extensively binds to CAA‐related vascular deposits,^30^ further accelerating amyloid clearance, which contributes to the development of ARIAs.^4^ There is the suggestion of a dose‐dependent relationship between CAA severity and risk of ARIA‐E.^31^ In this study, given the association of diffusion abnormalities consistent with ischemic stroke and ARIAs, most commonly involving the cortex or cerebellum, it could be hypothesized that AAT‐mediated removal or immune‐mediated engagement of vascular Aβ may increase the risk of local micro‐ischemia visible on DWI in a subset of patients. Underlying CAA may predispose these patients to microvascular injury, while perivascular cells may also contribute through dysregulated amyloid clearance. This may signify a differential pathophysiology of AAT‐related stroke, compared to common causes of silent brain infarction that are specific to patients being managed with AATs.

The CLARITY‐AD trial,^7^ which led to FDA approval for use of lecanemab in patients with early AD, reported rates of ARIA‐E and ARIA‐H detected on therapeutic MRI monitoring as safety endpoints. DWI was included in imaging protocols, but new areas of restricted diffusion consistent with ischemia were not a reported event within this study. The open‐label extension further did not report on stroke and/or DWI imaging abnormalities.^32^ Within the trial, 71% of ARIA‐E events occurred within the first 3 months of treatment,^7^ early in the treatment course as Aβ is cleared. Likewise, within our cohort, the median time to diagnosis of ischemia was 78 days, with 72.7% of events occurring within the first 3 months after the initiation of treatment. Given the significant association of ARIAs with diffusion abnormalities consistent with ischemia within our cohort, this temporal association is suggestive of a possible linked mechanism between ARIAs and these regions of cerebral and cerebellar ischemia.

Within our cohort of patients mostly over age 70, there were other mechanisms that could contribute to ischemic stroke. Extended cardiac monitoring was performed in 72.7% of patients with 25.0% of patients having detectable atrial fibrillation. Advancing age is not only the most important non‐modifiable risk factor for the development of AD, but also for atrial fibrillation.^33^ There was a non‐significant increased rate of atrial fibrillation in patients on lecanemab compared to placebo (0.7% vs. 0.3%) in the CLARITY‐AD trial,^7^ though there has been no evidence for a causal link. In patients with cerebral ischemia using AATs, it remains paramount to investigate vascular risk factors and common etiologies of stroke. Currently, therapeutic anticoagulation is not recommended for patients prescribed AATs^11^, ^34^ given increased risk of bleeding. In those with atrial fibrillation, left atrial appendage closure could be considered to reduce the risk of stroke and avoid long‐term anticoagulation.^9^ One patient in our cohort receiving lecanemab with new‐onset atrial fibrillation successfully received left atrial appendage closure with the goal of continuing therapy.^35^ The majority of patients within this cohort with stroke were started on antiplatelet medications, which appear to be well tolerated in patients on AATs.^34^

Appropriate use recommendations for lecanemab^11^ exclude patients with stroke or TIA within 12 months, and thus these associated diffusion abnormalities consistent with ischemia can impact subsequent therapy. The association with ARIAs and a hypothesis that some of these imaging abnormalities may have a shared mechanism with ARIAs raises questions about the clinical significance of these findings and management considerations. Current guidelines give clear instruction regarding ARIA management. For example, in asymptomatic ARIAs, lecanemab therapy may continue under monthly MRI monitoring with discontinuation of monthly MRI if the ARIA resolves or stabilizes.^11^ These regions of diffusion restriction in patients prescribed AATs have not been previously described in a large cohort; therefore, management strategies regarding continuation of lecanemab are unclear. In patients with a new indication for anticoagulation, such as atrial fibrillation, it would align with current use recommendations to hold therapy with lecanemab.^11^ If some of these ischemic lesions share a similarly mediated pathway as the ARIA, it could be hypothesized that existing protocols, once alternative etiologies are ruled out, would be reasonable to follow for therapeutic drug monitoring with lecanemab as it pertains to patients with ARIAs.

Our finding that patients who experienced stroke had lower mean baseline MoCA scores is consistent with real‐world evidence from memory clinics evaluating lecanemab use and further affirms a potential benefit of earlier diagnosis and treatment in symptomatic AD. A recent study reports that patients who developed symptomatic ARIAs had significantly lower baseline cognitive scores compared to those who did not develop ARIAs.^36^ Together, these findings raise the possibility that higher baseline cognitive impairment may be associated with increased vulnerability to vascular adverse events.

This study has multiple known limitations. The retrospective, observational, single‐center design could insert biases within our cohort. The low rate of ischemic lesions restricts the statistical power of conclusions, and thus these must be interpreted with caution. Given the observational nature of this study, the temporal associations cannot establish causality, and thus future work is needed to investigate these hypotheses. Given the frequency of MRI monitoring within this cohort, there also may be detection bias of clinically silent ischemia. Within the study, there was not uniform workup for stroke and thus incomplete workups may limit the determination of a hypothesized mechanism for each infarct.

In conclusion, this study of 261 patients with positive AD biomarkers managed on lecanemab shows 4.2% of patients were found to have a new region of restricted diffusion on DWI imaging consistent with stroke with median time to abnormality of 78 days. A history of diagnosis of ARIA was found to be significantly associated with diagnosis of stroke with restricted diffusion on DWI, and further found to be significant using a propensity‐matched analysis matching for age, sex, baseline MoCA, diagnosis, and number of *APOE* ε4 alleles. Higher than expected rates of cerebellar and cortical diffusion restriction and a significant association of ARIAs suggests the potential of a common mechanism between these regions of restricted diffusion and ARIAs. Larger, multi‐center registry studies could be used to further assess the association of ARIAs and new DWI lesions consistent with ischemia to better validate this association. Mechanistic studies incorporating advanced imaging, high‐resolution vessel wall imaging, quantitative susceptibility mapping, blood–brain barrier permeability imaging, and fluid biomarkers of vascular inflammation may further clarify whether these lesions represent immune‐mediated microvascular injury distinct from traditional ischemic mechanisms. This would allow further evaluation of these imaging abnormalities to assess long‐term risk and management strategies given the association with ARIAs to determine the clinical significance and imaging significance of these lesions.

## AUTHOR CONTRIBUTIONS

**Dylan Ryan**: Wrote the manuscript, collected data and conceived the study. **Michael W. Lutz**: Analzyed the data. **Tori Sides**: Collected data. **Richard O'Brien**: Conceived the study. **Kim Johnson**: Collected data, conceived the study, and edited the manuscript.

## CONFLICT OF INTEREST STATEMENT

There are no author conflicts of interest. Author disclosures are available in the supporting information.

## CONSENT STATEMENT

All human subjects have provided informed consent.

## Supporting information



Supporting Information

Supporting Information
