# Supplementary material for: Diffusion weighted imaging abnormalities and cerebral ischemia in a cohort of patients on lecanemab
Source: Alzheimers Dement. 2026 Apr 21;22(4):e71392. doi: 10.1002/alz.71392 (PMC13099582; doi:10.1002/alz.71392)
Supplement: Supplementary file 1 — Supporting Information [file ALZ-22-e71392-s002.docx]

**Supplemental Table 1: Statistical Comparisons of Demographic and Clinical Variables**

|  | **No stroke** | **stroke** | **test statistic** | **df** | **p value (significant difference between stroke and no stroke)** | **FDR p** | **Wilk Shapiro** | **p value** | **Statistical Test** |
| --- | --- | --- | --- | --- | --- | --- | --- | --- | --- |
| **N** | 250 | 11 |  |  |  |  |  |  |  |
| **Age, y, mean (SD)** | 74.3 (6.3) | 80.1 (3.6) | 24.7 | 1, 12.8 | 0.0003 | 0.005 | 0.97 | <0.0001 | t-test with Welch correction |
| **Sex  % Female** | 53% | 27% |  |  | 0.12 | 0.42 |  |  | Fisher exact, two-tailed |
| **Race** |  |  | 4.1 | 2 | 0.13 | 0.42 |  |  | Likelihood ratio |
| **White, N, %** | 230, 92% | 9, 82% |  |  |  |  |  |  |  |
| **Black** | 9, 4% | 2, 18% |  |  |  |  |  |  |  |
| **Other** | 11, 4% | 0, 0% |  |  |  |  |  |  |  |
| **E4 alleles** |  |  | 3.2 | 2 | 0.2 | 0.42 |  |  | Likelihood ratio |
| **0** | 32% | 45% |  |  |  |  |  |  |  |
| **1** | 56% | 55% |  |  |  |  |  |  |  |
| **2** | 12% | 0% |  |  |  |  |  |  |  |
| **Diagnosis - MCI, dementia** |  |  | 0.11 | 1 | 1 | 1 |  |  | Likelihood ratio |
| **MCI** | 166, 68% | 8, 73% |  |  |  |  |  |  |  |
| **AD** | 78, 32% | 3, 27% |  |  |  |  |  |  |  |
|  |  |  |  |  |  |  |  |  |  |
| **MOCA baseline** | 22 (2) | 19 (2) | 6.11 | 1 | 0.014 | 0.1 |  |  | ordinal logistic regression |
| **MMSE baseline** | 26 (2) | 26 (1) | 0.016 | 1 | 0.9 | 1 |  |  | ordinal logistic regression |
| **Fazekas score** | 1 (0) | 1 (1) | 1.67 | 1 | 0.2 | 0.42 |  |  | ordinal logistic regression |
| **Any ARIA** | 52, 21% | 6, 55% | 5.7 | 1 | 0.017 | 0.1 |  |  | Logistic regression, after propensity score matching |
| **HTN** | 136, 54% | 6, 55% | 0 | 1 | 1 | 1 |  |  | Fisher exact, two-tailed |
| **HLD** | 178, 71% | 10, 91% | 2.5 | 1 | 0.3 | 0.51 |  |  | Fisher exact, two-tailed |
| **DM** | 40, 16% | 0, 0% | 3.7 | 1 | 0.22 | 0.42 |  |  | Fisher exact, two-tailed |
| **AF** | 15, 6% | 2, 18% | 1.8 | 1 | 0.18 | 0.42 |  |  | Fisher exact, two-tailed |
| **BMI >30** | 56, 22% | 2, 18% | 0.11 | 1 | 1 | 1 |  |  | Fisher exact, two-tailed |
| **OSA** | 75, 30% | 2, 18% | 0.77 | 1 | 0.52 | 0.8 |  |  | Fisher exact, two-tailed |
| **Tobacco use** | 80, 32% | 4, 36% | 0.09 | 1 | 0.75 | 1 |  |  | Fisher exact, two-tailed |
| **Hx CVA/TIA** | 27, 11% | 1, 9% | 0.034 | 1 | 1 | 1 |  |  | Fisher exact, two-tailed |
